# Nucleosome DNA unwrapping does not affect prototype foamy virus integration efficiency or site selection

**DOI:** 10.1371/journal.pone.0212764

**Published:** 2019-03-13

**Authors:** Randi M. Mackler, Nathan D. Jones, Anne M. Gardner, Miguel A. Lopez, Cecil J. Howard, Richard Fishel, Kristine E. Yoder

**Affiliations:** 1 Department of Cancer Biology and Genetics, The Ohio State University College of Medicine, Columbus, OH, United States of America; 2 Department of Chemistry and Biochemistry, The Ohio State University, Columbus, OH, United States of America; University of St Andrews, UNITED KINGDOM

## Abstract

Eukaryotic DNA binding proteins must access genomic DNA that is packaged into chromatin *in vivo*. During a productive infection, retroviral integrases (IN) must similarly interact with chromatin to integrate the viral cDNA genome. Here we examine the role of nucleosome DNA unwrapping in the retroviral integrase search for a target site. These studies utilized PFV intasomes that are comprised of a tetramer of PFV IN with two oligomers mimicking the viral cDNA ends. Modified recombinant human histones were used to generate nucleosomes with increased unwrapping rates at different DNA regions. These modifications included the acetylmimetic H3(K56Q) and the chemically engineered H4(K77ac, K79ac). While transcription factors and DNA damage sensors may search nucleosome bound DNA during transient unwrapping, PFV intasome mediated integration appears to be unaffected by increased nucleosome unwrapping. These studies suggest PFV intasomes do not utilize nucleosome unwrapping to search nucleosome targets.

## Introduction

Eukaryotic biology is dependent on proteins interacting with DNA in the context of chromatin. An enduring mystery in retrovirology is the criteria used by the viral protein integrase (IN) to choose an integration site in host chromatin. Integration of a reverse transcribed viral complementary DNA (cDNA) into the host genome is required for replication [1]. Integration site selection is not random and appears to be influenced by multiple factors including the port of nuclear entry, chromatin features, local DNA sequence and, in some cases, host cofactors for integration [2–8]. Over twenty years ago, human immunodeficiency virus 1 (HIV-1) and murine leukemia virus (MLV) integration were found to favor DNA wrapped in nucleosomes *in vitro* and *in vivo* [9–12]. Since those initial studies, host proteins that serve as integration cofactors have been identified, including LEDGF/p75 for HIV-1 and BET proteins for MLV [2, 7, 13, 14]. Although host cofactors of integration had not been described at the time of the earliest chromatin studies, several key observations of retroviral integration with nucleosomes were made. Not surprisingly, integration sites were overwhelmingly confined to the exposed DNA regions of nucleosomes [15, 16]. Combined with studies of intrinsically and physically bent DNA, these observations are consistent with the conclusion that bent or distorted DNA is a preferred target for retroviral integration [9, 10, 15].

Chromatin is principally comprised of nucleosomes, which consist of ~147 bp of DNA wrapped around an octamer of histone proteins H2A, H2B, H3, and H4. Nucleosomes may be assembled *in vitro* with recombinant human histones expressed in bacteria [17]. Several model nucleosome positioning sequence (NPS) DNAs have been characterized [18–21]. Systematic evolution of ligands by exponential enrichment (SELEX) was used to generate the synthetic 601 NPS which yields highly stable nucleosomes [22]. The extensive biophysical studies of 601 NPS-derived nucleosomes, including high resolution structures, afford a substantial context for analysis of retroviral integration [21, 23–29]. Specifically, the dynamics of 601 nucleosome DNA unwrapping have been quantified and modeled using a free energy landscape [25].

While the 601 NPS produces a highly stable and well-positioned nucleosome, the 20 bp on each end that constitute the entry-exit regions have been shown to rapidly fluctuate or “breathe” by transient unwrapping and re-wrapping of the NPS DNA [28, 30]. These fluctuations in DNA wrapping allow transcription factors or DNA damage sensors to access sites that are otherwise occluded by the binding interface with the histone octamer [24, 27, 28, 30, 31]. More internal sequences, including the central nucleotides of the NPS termed the dyad, exhibit significantly less breathing and less accessibility [32]. Importantly, unwrapping of DNA from the entry-exit region to the dyad is irreversible and results in disassembly of the nucleosome [29, 33–35]. A region between the dyad and entry-exit was found to be associated with loss of rDNA silencing (LRS) based on genetic studies in yeast [36]. As might be predicted, restriction enzymes and the LexA transcription factor are better able to bind the entry/exit regions than the LRS and dyad regions [24, 27, 28].

The functional retroviral integration complex, or “intasome”, of prototype foamy virus (PFV) is a tetramer of IN with two viral cDNA ends which may be mimicked by two DNA oligomers [37]. A cryo-electron microscopy (cryo-EM) structure showed the PFV intasome bound at a single site on a mononucleosome [38]. Previous studies of HIV-1 integration identified the same nucleosome site, as well as several additional positions around the nucleosome [9]. It is not known how the intasome selects an integration site on a nucleosome. Single molecule studies showed that PFV intasomes perform an extensive one-dimensional (1D) rotation coupled diffusion search on naked linear DNA [39]. The role of rotation coupled diffusion during the retroviral intasome search of chromatin is also unknown. One hypothesis is that the PFV intasome takes advantage of transient nucleosome DNA unwrapping and slides to an internal site. As the NPS DNA rewraps, the intasome is effectively trapped and completes the integration reaction.

Here we examined the integration of PFV intasomes at physiological ionic conditions into 601 mononucleosomes reconstituted with recombinant human histones. We observed four major integration sites, including the site identified by cryo-EM studies with the D02 nucleosome. The integration sites on these unmodified nucleosomes were proximal to known core histone acetylation sites that increase the NPS DNA unwrapping rate. We engineered these histone acetylmimetics or acetylations to evaluate the role of increased unwrapping on PFV integration efficiency and site selection. We determined that nucleosome unwrapping or instability does not alter PFV integration. These results suggest that PFV intasomes do not search chromatin by sliding on transiently unwrapped DNA, but more likely by 3D diffusion with limited rotation coupled diffusion on exposed helices.

## Materials and methods

### DNA substrates

The 145 base pair (bp) D02 NPS was a synthetic double stranded DNA gBlock (Integrated DNA Technologies). The D02 gBlock was PCR amplified with forward primer 5’ GGCTGTGTTTGTATCAAGTTACC 3’ and reverse primer 5’ TGTCCAGGTTCTCCCTGT 3’. The PCR product was subcloned to pGemT easy (Promega). D02 NPS DNA for nucleosome assembly was PCR amplified from the plasmid with the same primers. The 147 bp Cy5-labeled 601 nucleosome positioning sequence (NPS) was PCR amplified from pDrive-601 with forward primer 5’ CTGTAGAATCCCGGTGCCGAGGCCGCT 3’ and reverse primer 5’ ACAGGATGTATATATCTGACACGTGCCTGGA 3’. For both NPS DNAs, the forward primer was fluorescently labeled with Cy5-NHS ester (GE Healthcare) at the fourth base from the 5’ end at an amino modified thymine (Integrated DNA Technologies). Labeled oligonucleotides were purified by reverse phase HPLC with a C18 Poroshell 120 column (Agilent Technologies). Following PCR, Cy5 labeled NPS DNA was purified by ion-exchange HPLC with a Gen-Pak Fax column (Waters).

DNA oligomers mimicking the PFV U5 end were oKEY616 5’ ATTGTCATGGAATTTTGTATATTGAGTGGCGCCCGAACAG 3’ and oKEY675 5’ CTGTTCGGGCGCCACTCAATATACAAAATTCCATGACA 3’ (Integrated DNA Technologies). The oligomers were annealed and purified as described [40].

### Nucleosomes

**Fig 1:**
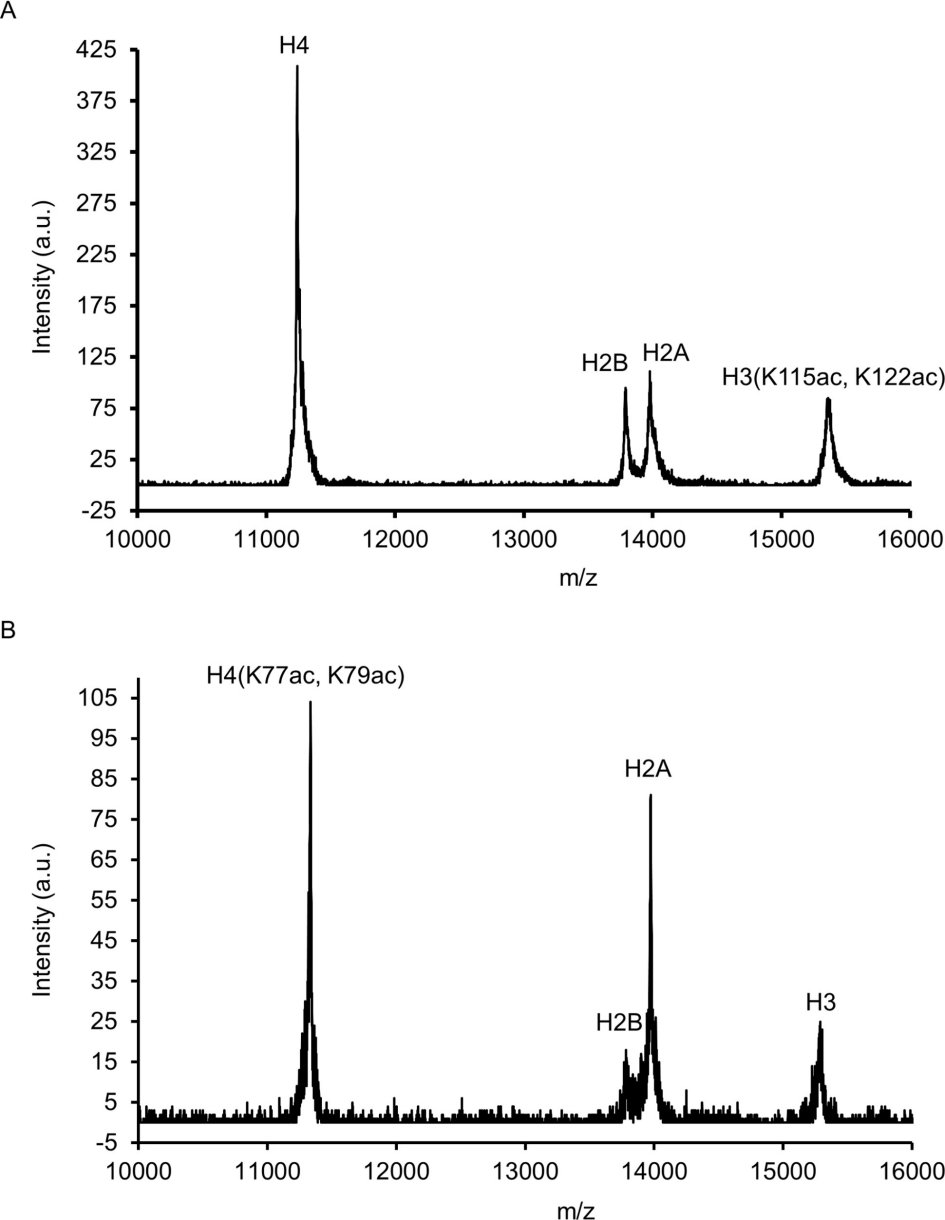
Mass spectrometry of acetylated histones. Acetylation of histones generated by EPL was confirmed by mass spectrometry. **A.** Representative mass spectra for H3(K115ac,K122ac). Expected m/z 15356, observed m/z 15355. **B.** Representative mass spectra for H4(K77ac,K79ac). Expected m/z 11321, observed m/z 11324.

**Fig 2:**
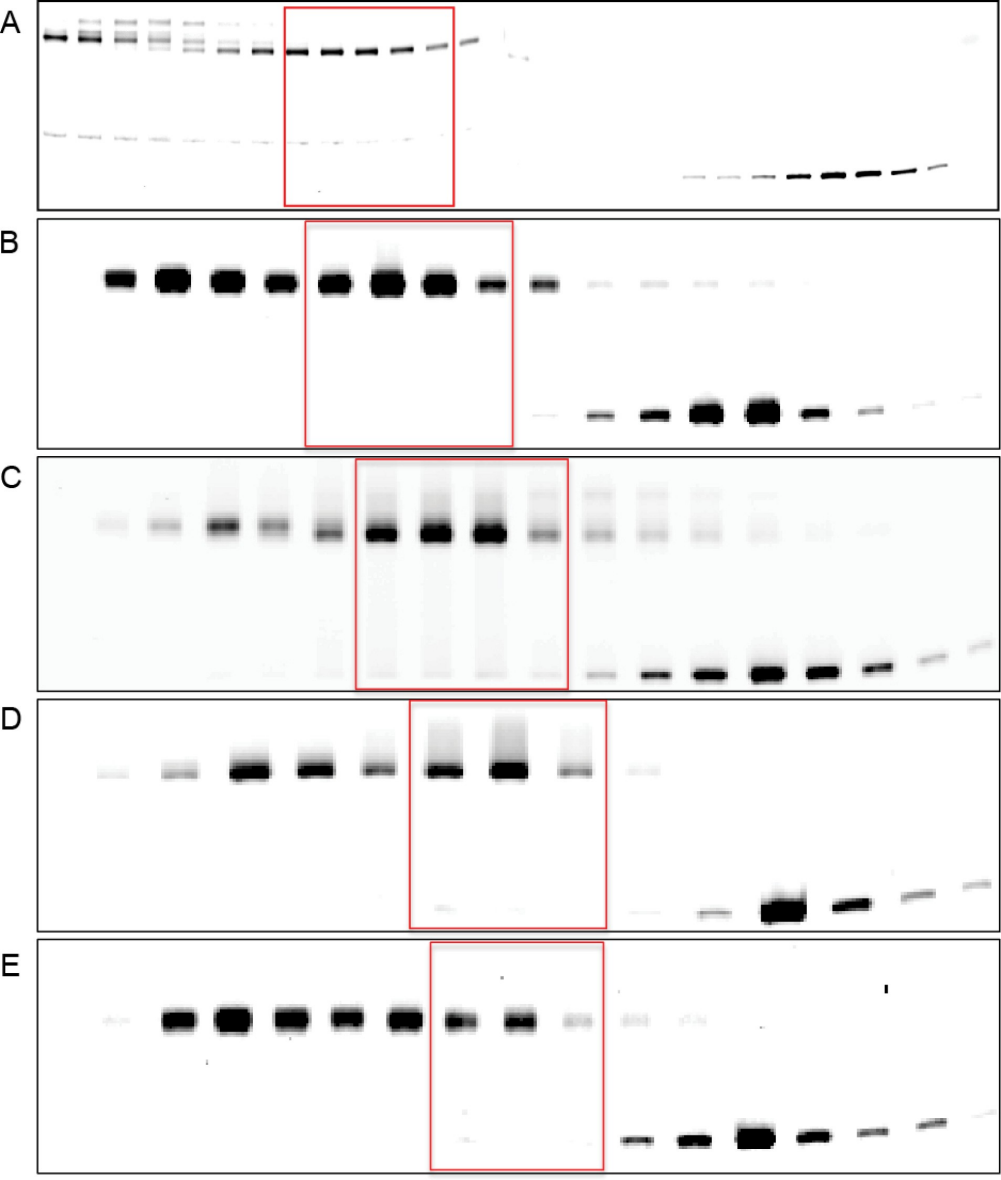
Native PAGE analysis of sucrose gradient fractions after nucleosome reconstitution. Histone octamers were reconstituted with Cy5 labeled D02 or 601 NPS DNA and subjected to sucrose gradient velocity centrifugation. Sucrose gradient fractions were analyzed by native PAGE. Fractions containing mononucleosomes without contaminating free DNA or excess histone proteins were combined and concentrated, red boxes. **A.** unmodified D02 nucleosomes, **B.** unmodified 601 nucleosomes, **C.** 601 H3(K56Q) nucleosomes, **D.** 601 H4(K77ac,79ac) nucleosomes, and **E.** 601 H3(K115ac,122ac) nucleosomes.

Unmodified, recombinant human histones H2A or H2A(K119C), H2B, H3, H3(K56Q), and H4 were expressed and purified as described [19]. Histones H3(K115ac, K122ac) and H4(K77ac, K79ac) were produced by expressed protein ligation as described [39, 41, 42]. The synthetic acetylations were confirmed by mass spectrometry analysis (Fig 1). Octamers were refolded at equimolar histone concentrations and purified by Superose 12 10/300 (GE Healthcare) size exclusion chromatography in 10 mM Tris-HCl pH 7.5, 2 M NaCl, 1 mM EDTA. Nucleosomes were reconstituted with Cy5 labelled 145 bp D02 DNA or 147 bp 601 DNA and histone octamer at a 1:1 molar ratio by double dialysis against 5 mM Tris-HCl pH 7.5, 0.5 mM EDTA, 1 mM benzamidine [43]. The products were separated by sucrose gradient velocity centrifugation [43]. Gradient fractions were analyzed by separation on a native polyacrylamide gel electrophoresis (PAGE) and imaged using a Typhoon 9410 variable mode fluorescent imager (GE Healthcare) or a Sapphire Biomolecular Imager (Azure Biosystems) (Fig 2). Fractions with fluorescent NPS DNA bound by histone octamer were combined. The sample buffer was exchanged to 5 mM Tris-HCl pH 7.5, 0.5 mM EDTA and nucleosomes were concentrated with Amicon Ultra centrifugal filters (EMD Millipore). Nucleosomes were stored at 4°C.

### PFV integration

PFV intasomes were assembled and purified as previously described [40, 44, 45]. All experiments were performed with at least two independent PFV intasome purifications. Integration reactions contained 10 mM Bis-tris propane pH 7.5, 110 mM NaCl, 5 mM MgSO_4_, 4 μM ZnCl_2_, and 10 mM DTT, the indicated concentration PFV intasomes, and 15 ng NPS DNA in nucleosomes in a final volume of 15 μL. Reactions were incubated at 37°C for 5 min and stopped with 0.5% SDS, 1 mg/mL proteinase K, and 20 mM EDTA. Reactions were incubated at 55°C for 1 hr. Products were separated by denaturing PAGE and scanned with a Typhoon 9410 variable mode fluorescent imager (GE Healthcare) or a Sapphire Biomolecular Imager (Azure Biosystems).

Denaturing PAGE gel analysis was performed using BioNumerics 7.6 (Applied Maths). Molecular weight standards (GeneScan 120 LIZ Size Standard, ThermoFisher Scientific) were fit to an exponential decay. The molecular weight of each integration band (± 3 nucleotides (nt)) was calculated relative to the molecular weight ladder. Total integration efficiency was determined by subtracting the fraction of unreacted NPS from the normalized total signal. The intensity profile of each lane was used to quantify the relative amount of DNA in each band (BioNumerics). The area under each peak of the intensity profile was calculated as a fraction of the total lane intensity (OriginPro 9.1, OriginLab). These fractions of the total intensity are relative measures of integration efficiency. Individual peaks in a band cluster could not be distinguished individually as a result of overlapping pixel density. The data are presented as averages with error bars indicating the standard deviation (s.d.) of at least three independent experiments. Data was analyzed by paired t test and ANOVA.

## Results

### PFV integration assays with a natural NPS

**Fig 3:**
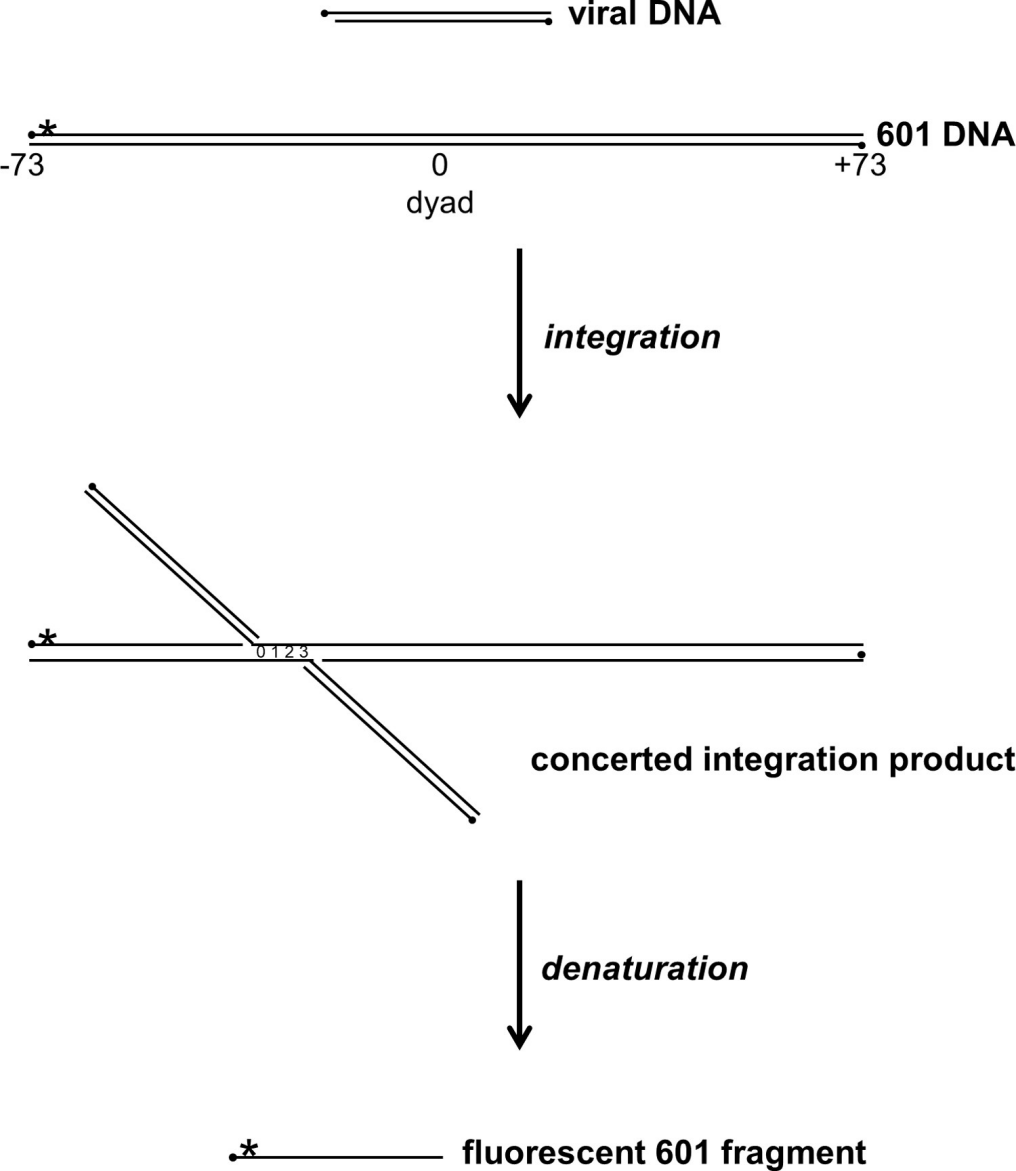
Illustration of PFV integration to a linear NPS target DNA. The PFV viral DNA is added to nucleosomes. The 601 NPS DNA is 147 bp DNA numbered from the dyad (0) to ±73 (shown). The D02 NPS DNA is 145 bp DNA similarly numbered from the dyad (0) to ±72 (not shown). Black circles indicate 5’ ends. Asterisk indicates a Cy5 fluorescent moiety. During integration the viral DNA 3’ end is covalently joined to the target DNA. Two viral DNAs are joined separated by 4 bp during concerted integration. The NPS DNA is broken by the integration event. Denaturation of integration products liberates a fluorescently labeled fragment that indicates one site of viral DNA joining.

A tetramer of recombinant PFV integrase (IN) and two retroviral donor DNA oligomers mimicking the viral DNA ends (vDNA) may be assembled and purified as a functional intasome complex [37, 40]. PFV intasomes covalently join the vDNA ends to a target DNA in two kinetically distinct strand transfer reactions separated by 4 bp of target DNA, termed concerted integration (Fig 3). PFV intasome concerted integration into a circular target DNA results in a linear product with vDNA at the termini [46]. Concerted integration to an NPS DNA will generate two fragments, each with a 4 base gap at the junction of vDNA and NPS DNA. DNA gaps may significantly alter mobility on a native gel precluding accurate determination of integration sites [47]. In order to more accurately determine the sites of integration on a nucleosome, the reaction products were analyzed by denaturing PAGE. Integration to Cy5 labeled nucleosome DNA will generate a break on the labeled strand (Fig 3). The length of this band indicates the site of a strand transfer event. The central base pair (bp) of an NPS is numbered 0 and termed the dyad (Fig 3). Left (5') and right (3') flanking sequences are numbered outward from the dyad, negative and positive, respectively. The mirror symmetry of the histone octamer proteins is reflected in the symmetry of the DNA numbering, such as -36 and +36 (referred to as ±36). In some cases, intasomes will only join one vDNA end to the target DNA, termed half site integration. Recombinant PFV intasomes have been shown to readily perform concerted integration with relatively few half site integration events [39, 46]. This denaturing PAGE analysis measures total integration activity, including both concerted and half site integration events.

Several NPS DNA sequences have been described for the reconstitution of nucleosomes *in vitro* [48]. Naturally occurring NPS sequences, such as one derived from the *Xenopus borealis* 5S rDNA sequence, display multiple overlapping positions of the histone octamer particularly under physiologic ionic conditions [48, 49]. In order to definitively map integration sites, the position of the NPS DNA relative to the histone octamer must be static. An elegant cryo-EM structure of the PFV intasome bound to a single site on a mononucleosome has been reported [38]. This structure employed a natural NPS derived from HeLa cells, termed D02 [38]. Although natural NPS DNAs are known to slide on the histone octamer, the visualization of a single PFV intasome binding site suggested this NPS could be stable. Mononucleosomes were reconstituted from recombinant human histone octamer and D02 NPS DNA labeled on one strand with a Cy5 fluorophore. Cy5 labeled D02 nucleosomes were purified by sucrose gradient and analyzed by native gels (Fig 2A). Nucleosomes displayed reduced mobility and were readily distinguishable compared to free NPS DNA. Nucleosome assembly *in vitro* may also result in spurious products that include excess histone proteins. These nucleosomes appeared as a secondary peak of slightly higher molecular weight compared to the correct octamer (most apparent in Fig 2A and 2C). Sucrose fractions that were free of naked NPS DNA and higher molecular weight contaminants were combined and used for integration studies.

**Fig 4:**
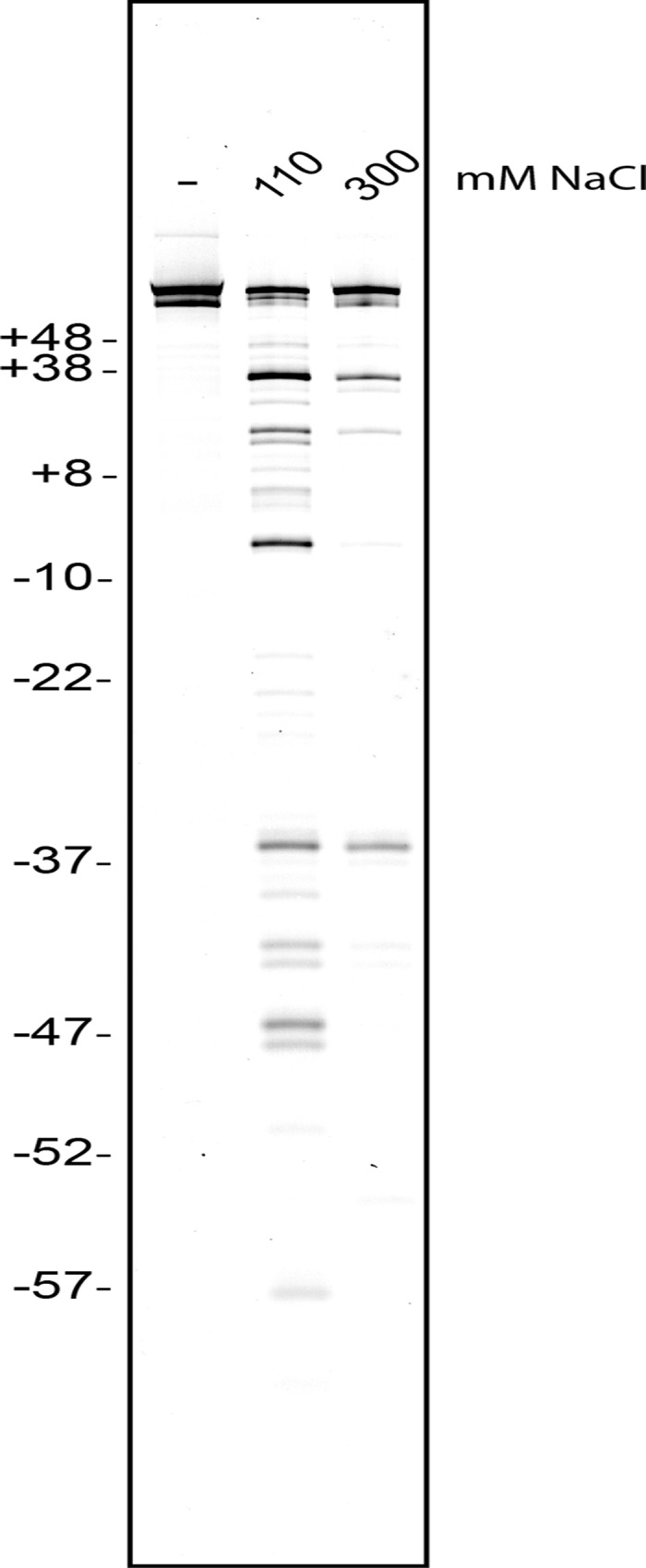
PFV integration into D02 nucleosomes. PFV intasomes were added to Cy5 labeled D02 NPS nucleosomes with unmodified histones in the presence of 110 mM or 300 mM NaCl. Integration products were resolved by denaturing PAGE and imaged for Cy5 fluorescence. The PFV intasome concentration was 26 nM. 145 bp D02 nucleosome substrate without PFV intasomes (-). Marker sizes are shown as nucleosome position numbers relative to the central dyad, left side. Representative gel of at least three independent experiments with at least two independent preparations of PFV intasomes. Complete gel images are shown in S1 Fig.

PFV intasomes were added to the D02 nucleosomes at two NaCl concentrations: physiologically relevant 110 mM NaCl and non-physiological 300 mM NaCl (Fig 4). PFV intasomes do not lose activity in the presence of higher ionic concentrations [50]. A cryo-EM structure of the PFV intasome bound to a D02 nucleosome was achieved in the presence of 290 mM NaCl [38]. PFV integration products measured by denaturing PAGE revealed similar results to the cryo-EM structure with integration at the symmetric sites of -36 and +36 (Fig 4). Compared to the higher salt conditions, more integration sites were observed under physiologically relevant ionic conditions. Comparing the integration sites to the structures of nucleosomes similar to 145 bp D02 NPS nucleosomes indicate that several of these observed integration sites are at buried DNA regions and predicted to prevent integration [21, 51, 52]. To account for these unexpected integration sites, the D02 NPS must experience fluctuations of rotational and/or translational DNA positioning. Thus the D02 sequence appears to behave similarly to other naturally occurring NPS DNA sequences under physiological conditions, making it not amenable to definitively map integration sites at physiological ionic strength.

### PFV integration assays with a synthetic NPS stable under physiologic conditions

**Fig 5:**
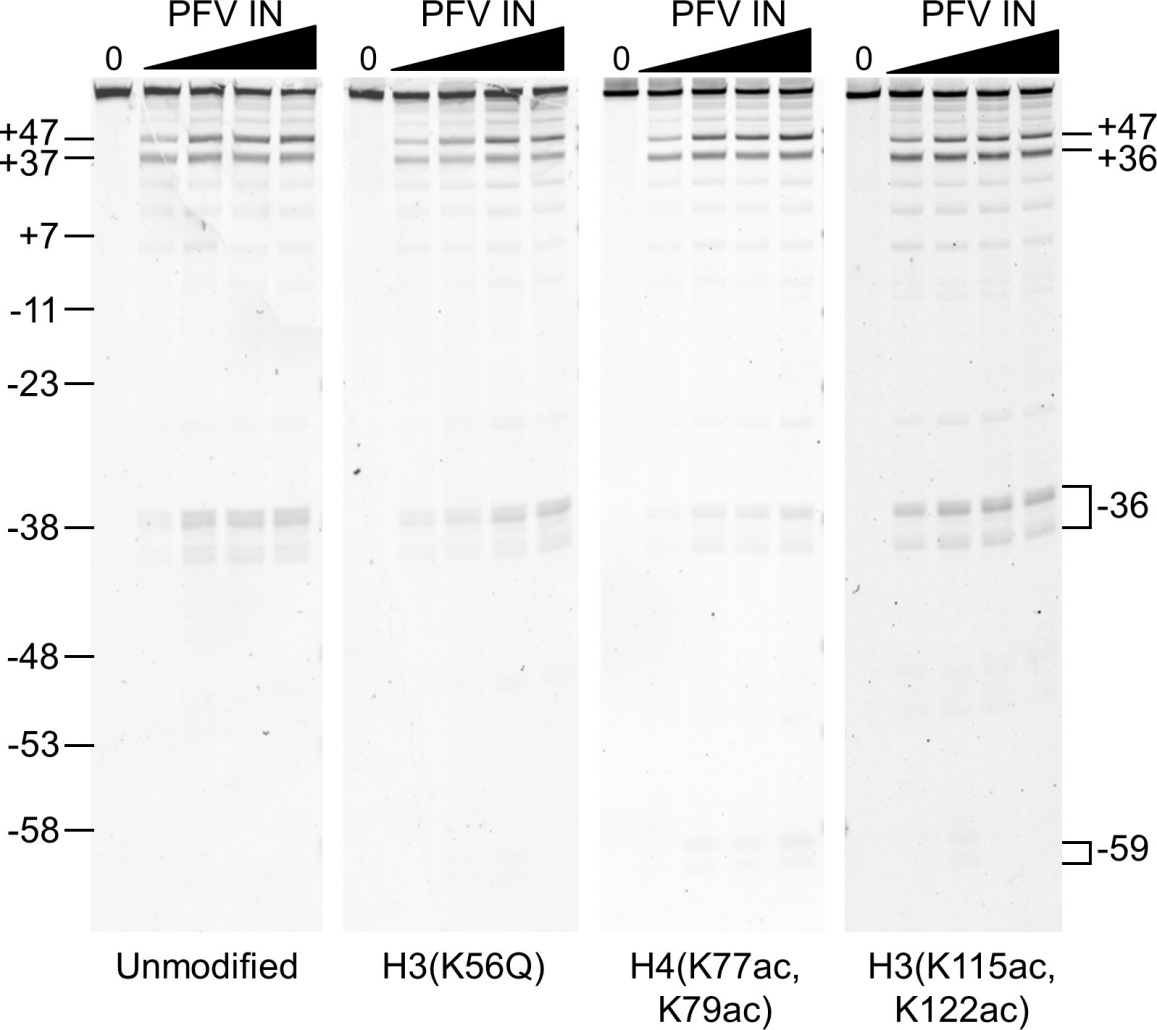
PFV integration into nucleosomes with histone PTMs affecting unwrapping and stability. PFV intasomes were added to Cy5 labeled 601 NPS nucleosomes with unmodified histones, H3(K56Q), H4(K77ac,K79ac), or H3(K115ac,K122ac). Integration products were resolved by denaturing PAGE and imaged for Cy5 fluorescence. The PFV intasome concentrations were 7 nM, 13 nM, 20 nM, and 26 nM, black triangles. 147 bp nucleosome substrate without PFV intasomes (-). Marker sizes are shown as nucleosome position numbers relative to the dyad, left side. Integration sites, right side. Representative gels of at least three independent experiments with at least two independent preparations of PFV intasomes and nucleosomes are shown. Complete gel images are shown in S1 Fig.

The 601 NPS was engineered specifically to stably position the histone octamer relative to the DNA at or below physiologically relevant salt concentrations [22]. A titration of PFV intasomes was added to Cy5 labelled 601 NPS nucleosomes (Fig 5). Multiple fragments were observed near nucleosome positions -59 and -36. Bands were also seen at +36 and +47, which may similarly be several fragments that are not resolved in this region of the gel. Other faint bands are observed at <2% of the total fluorescent signal in the reaction. Due to their low abundance, these bands were excluded from further analysis. Mapping the observed strand scissions to the 601 nucleosome structure (PDB 3LZ0) indicates integration sites are located on the outer DNA surface, not occluded by the histone octamer or the adjacent DNA gyre [21]. This is consistent with previous reports that integration into the dyad region of the nucleosome substrates is disfavored [9, 10].

The major observed integration sites are proximal to known core histone acetylation post-translational modifications (PTMs) that increase the unwrapping rate of the NPS. Specifically, the -59 cluster is in the entry-exit region of the nucleosome and near H3(K56). The +36 and -36 integration sites in the LRS region are near H4(K77,K79). We investigated the hypothesis that increased unwrapping of the NPS via engineered PTMs could increase PFV integration at these sites.

### PFV integration to modified mononucleosomes

Recombinant human histone proteins expressed in bacteria have no PTMs. Modified histones were engineered and incorporated into nucleosomes. Acetylated histones were engineered by expressed protein ligation (EPL). H3(K56Q) is a mimetic of acetylated lysine known to increase NPS DNA unwrapping at both entry-exit regions of a 601 nucleosome [43]. Mononucleosomes generated with H3(K56Q) were directly compared to H3(K56ac) and displayed identical nucleosome DNA binding dynamics [43]. At other nucleosome locations, a glutamine substitution does not faithfully recapitulate acetylated lysine [41]. For example, direct comparison revealed significant differences in nucleosome DNA binding dynamics by glutamine at H3(K115) and H3(K122) or EPL acetylations at these sites [abbreviated as H3(K115ac, K122ac)] [41]. H3(K115ac, K122ac) is located at the nucleosome dyad, a region of the nucleosome where only one DNA gyre is present and has the highest affinity for the histone octamer. H4(K77ac, K79ac) enhances the unwrapping of the NPS DNA at the LRS regions [29]. The histone acetylations were confirmed by mass spectrometry (Fig 1). The modified histone proteins were assembled into nucleosomes with Cy5 labeled 601 DNA and purified by sucrose fractionation (Fig 2).

**Fig 6:**
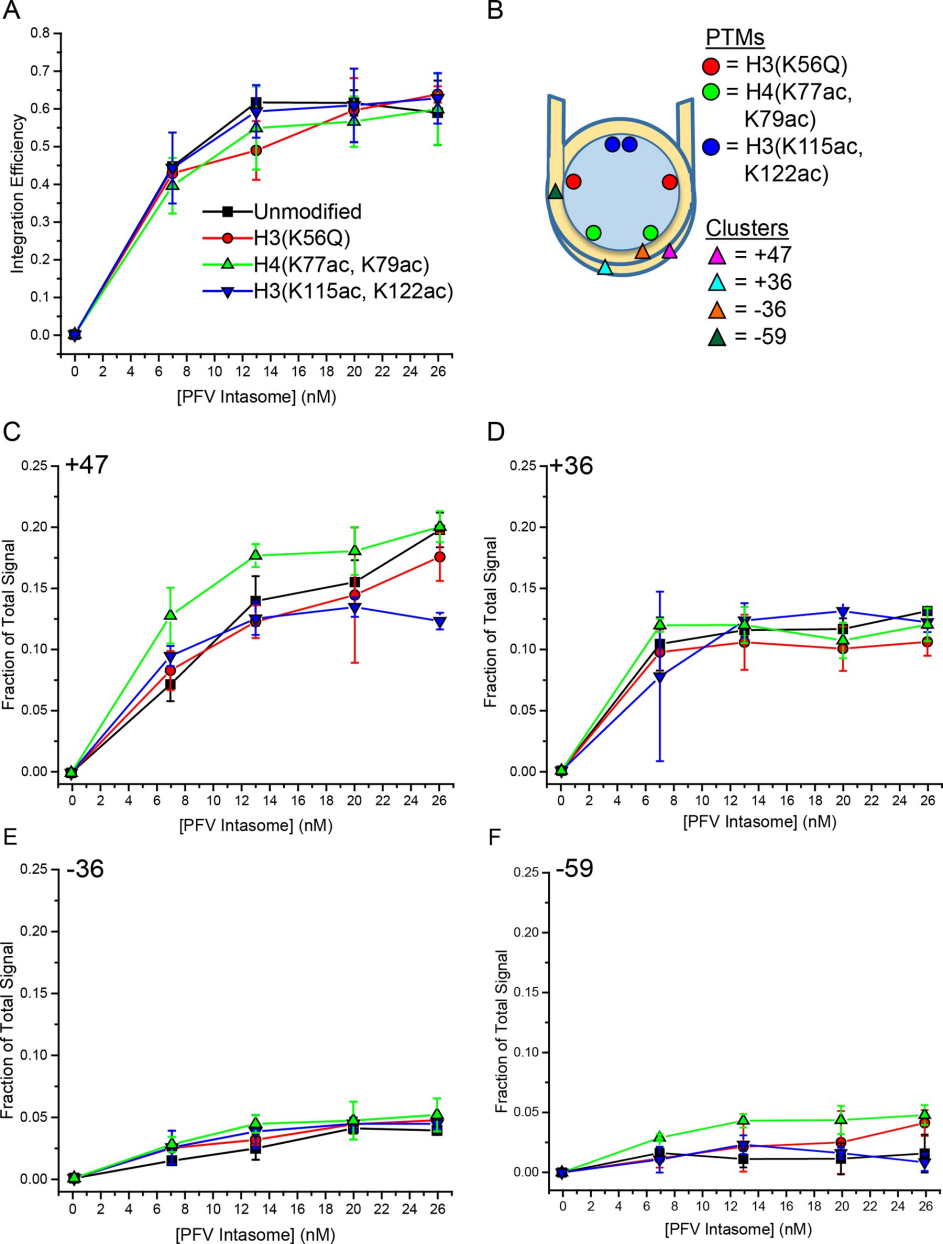
Quantitation of PFV integration into nucleosomes with core histone acetylations. **A.** Total integration activity of PFV intasome titrations added to Cy5 labeled nucleosomes with unmodified histones, H3(K56Q), H4(K77ac,K79ac), or H3(K115ac,K122ac). **B.** Nucleosome cartoon indicating the relative locations of histone PTMs and integration sites. Integration activity at each major site or cluster of sites: **C.** +47, **D.** +36, **E.** -36, and **F.** -59. Integrations to modified nucleosomes are compared to unmodified nucleosomes. Error bars indicate the standard deviation between at least three independent experiments with at least two PFV intasome preparations and two nucleosome preparations. Paired t test and ANOVA analysis indicate no significant differences. Minimal data sets of values depicted in the graphs are listed in S1 Table.

PFV intasomes were added to Cy5 labeled 601 nucleosomes containing three different histone acetylation sites (Figs 5 and 6). The addition of PFV intasomes decreased the apparent amount of full length 601 DNA. This reduction was interpreted as the total integration efficiency. Total integration into H3(K56Q), H4(K77ac, K79ac), or H3(K115ac, K122ac) nucleosomes was not significantly different than unmodified nucleosomes when analyzed by either t test or ANOVA (all p values >0.01, Fig 6A).

Although the total integration to nucleosomes was not affected by PTMs, the integration site choice could be altered. Integration site -59 in the nucleosome entry-exit region is in close proximity to H3(K56Q) (Fig 6B). H3(K56Q) increased LexA binding to a site in the 601 NPS entry-exit region 3 fold compared to unmodified nucleosomes in the presence of 130 mM NaCl [28]. Thus these nucleosomes are partially unwrapped at the entry-exit region. The increased unwrapping associated with this PTM extends from NPS ±73 DNA ends to ±47 [53]. We predicted that H3(K56Q) could affect PFV integration efficiency at the -59 cluster and possibly display minor effects on the +47 cluster. However, there was no change of integration efficiency at any site with H3(K56Q) nucleosomes (Figs 5 and 6).

PFV integration at ±36 was first reported by a cryo-EM structure and reproduced here with D02 and 601 nucleosomes [38] (Figs 4 and 5). The ±36 integration sites are in the LRS region near H4(K77ac, K79ac) (Fig 6B). H4(K77ac, K79ac) enhances unwrapping [29]. This unwrapping extends further from the ends of the NPS DNA than H3(K56Q), to approximately ±24 [53]. If NPS DNA unwrapping is important for PFV integration, then H4(K77ac, K79ac) should impact integration at -36, +36, and to a greater extent +47. However, analysis of PFV integration to H4(K77ac, K79ac) nucleosomes showed no difference in integration efficiency at any site (Figs 5 and 6).

We also evaluated nucleosomes with H3(K115ac, K122ac) to evaluate the effect of nucleosome stability on PFV integration (Fig 5). These modifications reduce the overall stability of the nucleosome without affecting unwrapping of the NPS DNA [41]. This PTM does not alter DNA accessibility at the dyad. Instead, this PTM is associated with increased histone dissociation [29]. We considered H3(K115ac, K122ac) could affect overall PFV integration efficiency or alter the integration site choice. However, PFV integration to H3(K115ac, K122ac) nucleosomes was not different from unmodified nucleosomes (Figs 5 and 6).

Integration showed no significant difference between any of the modified nucleosomes and unmodified nucleosomes (p>0.01). Paired t tests indicated that two individual points were significantly different from unmodified nucleosomes: integration site +47 at 26 nM PFV intasome with H3(K115ac,K122ac) (p = 0.004) and integration site -59 at 13 nM PFV intasome with H4(K77ac,K79ac) (p = 0.003). The site-specific integration efficiency data was further analyzed by ANOVA to thoroughly test the null hypothesis. This analysis determined all p values as >0.05, confirming that there is no significant difference between any of the modified nucleosomes and unmodified nucleosomes. Together, these integration studies into nucleosomes with specific histone PTMs suggest that the integration preference with 601 nucleosomes is not due to increased unwrapping kinetics. These observations appear to significantly limit the possible mechanisms for PFV intasome interrogation of a nucleosome target.

## Discussion

We have used recombinant human histones with specific PTMs that increase NPS unwrapping to dissect the mechanism of PFV intasome target search. We also tested PFV integration to a naturally occurring NPS derived from HeLa cells, but found that under physiological conditions the D02 NPS is not stably positioned relative to the histone octamer. In contrast, the synthetic 601 NPS was designed to remain stable at or below physiologically relevant ionic concentrations [22]. Although the 601 NPS has enhanced stability compared to natural NPS sequences, 601 displays predictable nucleosome dynamics when present in murine hepatocytes *in vivo* [54]. As previously shown with other retroviral integrases, PFV intasomes showed a preference for exposed DNA helices and significantly distorted regions of the NPS DNA. We identified four major sites in 601 nucleosomes that exhibited a cluster of 2–5 integration events. These observations suggest a limited search that is associated with integration events at these particular exposed helices of nucleosome DNA. The use of specific histone acetylation PTMs or mimetics here suggests that increased unwrapping rate or decreased nucleosome stability have no effect on PFV integration efficiency or target site selection.

An obvious question is why integration occurs at the symmetric sites ±36, but not ±59 or ±47. In addition, there is integration at both ±36, but +36 is favored compared to -36. The extensive biophysical data available for the 601 NPS offers some insights [26, 35]. The 601 NPS sequence is not symmetric and there are significant observed differences between the left and right halves. The left half of 601 DNA is more flexible than the right half [26]. This flexibility allows for stronger binding to the histone octamer. The right half, which includes the +36 and +47 sites, is a more rigid sequence and has weaker binding to the histone octamer. More force is required to disrupt the left half of the 601 nucleosome compared to the right half [35]. The cryo-EM image of the PFV intasome bound to the D02 NPS nucleosome suggested that the NPS DNA must pull away from the histone octamer [38]. The strong binding of the left half of the 601 NPS might prevent the dissociation of NPS DNA from the octamer and prevent integration at -47 and reduce integration at -36 relative to +36. The more weakly bound right half of the 601 NPS DNA empirically appears to readily allow integration at +47 and enhanced integration at +36.

These biophysical observations concerning the strength of NPS binding to the histone octamer did not apply to the entry-exit regions, extending from ±73 to approximately ±50 [26, 35]. It is notable that there is relatively little integration to -59 in the entry-exit region. In this case the sequence preference of integrase may offer an explanation [55, 56]. Integration strand scissions were apparent at -59 and -60. Both of these integration sites have a favored nucleotide at both strand transfer junctions. However, sequences at +59 and +60 do not display similarity to the PFV integrase sequence preference. The +60 sequence has no similarity and the +59 sequence would only have one base in common with the integrase preference. The lack of differences in the strength NPS binding at ±59 suggests that integration preference for -59 relative to +59 is due to DNA sequence preference.

The mechanism that PFV intasomes use to search DNA wrapped into nucleosomes appears to be distinct from that used by several transcription factors or DNA damage-sensing proteins. Because the wrapped DNA is partially occluded by the histone octamer, PFV intasomes cannot take advantage of their ability to search long distances of DNA by 1D rotation coupled diffusion. Our results with acetylated histones suggest that the PFV intasome does not diffuse on transiently unwrapped nucleosome DNA, which is more common in euchromatin. This data is in agreement with previous reports that PFV integration favors heterochromatin [38, 57]. The observation that other retroviruses, such as HIV-1, favor euchromatin may suggest that the intasome search mechanism is variable [58]. Further experiments with other retroviral intasomes will be necessary to determine the conservation of nucleosome search mechanism.

The PFV intasome search of nucleosomes has some features in common with several base excision repair glycosylases. Similar to PFV intasomes, uracil DNA glycosylase (UDG) has been shown to favor exposed regions of NPS DNA [59–61]. UDG prefers entry-exit regions and disfavors the dyad. This glycosylase is known to perform a 1D search by rotation coupled diffusion as well as intersegmental transfer which allows crosswise movement between exposed helices of the nucleosome-bound DNA [61–64]. PFV intasomes also use a 1D search with rotation coupled diffusion on linear DNA [39]. However, the search mechanics of the PFV intasome on nucleosome DNA is unknown. One possibility is that PFV intasomes search the nucleosome similarly to UDG by 3D diffusion or moving along the exposed helices of the NPS DNA. Further elucidation of the PFV and other retroviral intasome search mechanisms will likely require single molecule analytical resolution. However, our studies have begun to uncover the mechanisms underlying integrase searching of mononucleosome targets.

## Supporting information

S1 Fig Complete gel images for Figs 4 and 5. Black boxes correspond to the representative cropped gel images presented in Figs 4 and 5. Fig 5 gel images are shown in the order: Unmodified, H3(K56Q), H4(K77ac, K79ac), H3(K115ac, K122ac), left to right.
(TIF)

S1 Table Minimal data set for graphs shown in Fig 6. (XLSX)
