# Supplementary figures and images for: Nucleosome DNA unwrapping does not affect prototype foamy virus integration efficiency or site selection
Source: PLoS One. 2019 Mar 13;14(3):e0212764. doi: 10.1371/journal.pone.0212764 (PMC6415784; doi:10.1371/journal.pone.0212764)

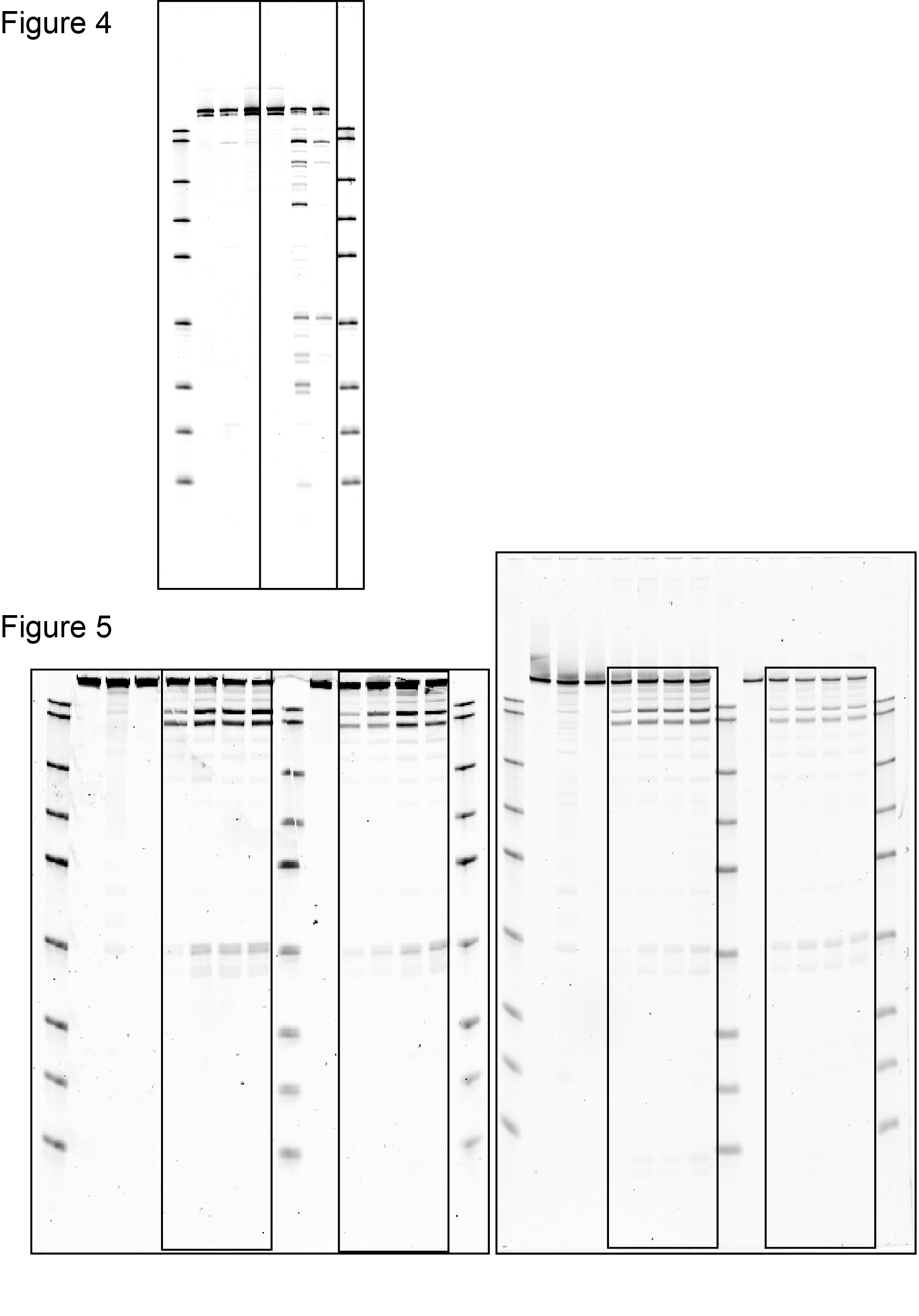

Supplement: S1 Fig — Black boxes correspond to the representative cropped gel images presented in Figs 4 and 5. Fig 5 gel images are shown in the order: Unmodified, H3(K56Q), H4(K77ac, K79ac), H3(K115ac, K122ac), left to right. (TIF) [file pone.0212764.s001.tif]
